# A Novel PKD1 Mutation in a Patient with Autosomal Dominant Polycystic Kidney Disease

**Published:** 2016-06-08

**Authors:** Javad Jamshidi, Hamed Naderi, Shaghayegh Taghavi, Babak Emamalizadeh, Hossein Darvish

**Affiliations:** 1 *Noncommunicable Diseases Research Center, Fasa University of Medical Sciences, Fasa, Iran.*; 2 *Department of Neurology, School of Medicine, Imam Khomeini Hospital AND Iranian Center of Neurological Research, Tehran University of Medical Sciences, Tehran, Iran.*; 3 *Department of Medical Genetics, School of Medicine, Shahid Beheshti University of Medical Sciences, Tehran, Iran.*

## Results

To the Editor,

Reporting novel mutations in genes related to human disorders along with their clinical complications can help prenatal diagnosis of the diseases through molecular genetic tests and also make it possible to predict the prognosis of the disease to some extends and consequently, the improved disease management. Herein we report a novel mutation in *PKD1* gene, which results in autosomal dominant polycystic kidney disease (APKD), one of the commonest severe renal disorders (1). The pathogenic *de novo* mutations of this gene have been reported to be about 10% of APKD cases (2). 

The patient is an Iranian 31-year old man who presented with no any familial history of the disease. None of his family members show the symptoms of the disease including his parents and a sister and a brother show the symptoms of the disease. The patient has a known history of hypertension since the age of 23, and experienced the first attack of renal colic at 27 years old. Sonography and CT scan imaging revealed enlarged and deformed kidneys with multiple cysts in different sizes, which show calcification in their walls and different sized stones. There was no sign of liver nor gastrointestinal involvement in imaging examinations until now. There was also no intracranial aneurysm detected in MRI imaging. No proteinuria was detected in urine analysis test and the blood creatinine was equal to 1.4 mg/dL. 

**Fig1:**
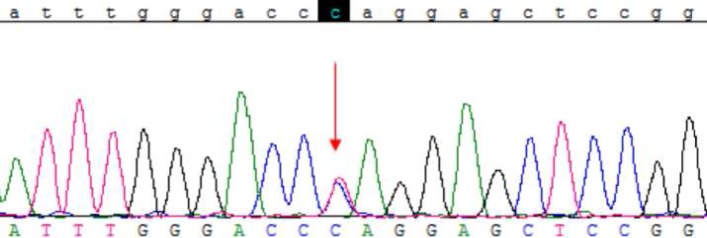
Location of the mutation. The substituted nucleotide (C>T) is indicated by red arrow. As it is clear the mutation is in heterozygous state.

As the patient was diagnosed for APKD, his *PKD1* and *PKD2* genes were sequenced to detect the possible mutation. A heterozygous mutation c.1789C>T (p.Gln597Ter) in Exon 9 of* PKD1* gene was detected (figure 1). This mutation has not been reported for its pathogenicity yet. The nonsense mutation leads to early termination of the translation, which is expected to affect the protein function. This mutation is possibly pathogenic for the patient, which is consistent with the clinical diagnosis. As there was no mutation in *PKD1* gene of his parents, it is considered as a *de novo* mutation. 

As most studies did not report a clear genotype-phenotype correlation between PKD1 mutations and the severity of the disease, reporting such mutations along with clinical phenotypes would be helpful to unravel the exact mechanism by which the PKD1 mutations cause various phenotypes. Here we reported a mutation which caused relatively mild APKD in our patient.
